# Vestibular rehabilitation with visual stimuli in peripheral vestibular disorders^☆^

**DOI:** 10.1016/j.bjorl.2015.05.019

**Published:** 2016-01-08

**Authors:** Andréa Manso, Mauricio Malavasi Ganança, Heloisa Helena Caovilla

**Affiliations:** Escola Paulista de Medicina, Universidade Federal de São Paulo (UNIFESP), São Paulo, SP, Brazil

**Keywords:** Rehabilitation, Postural balance, Dizziness, Nystagmus optokinetic, Tontura, Reabilitação, Nistagmo optocinético, Equilíbrio postural

## Abstract

**Introduction:**

Visual stimuli can induce vestibular adaptation and recovery of body balance.

**Objective:**

To verify the effect of visual stimuli by digital images on vestibular and body balance rehabilitation of peripheral vestibular disorders.

**Methods:**

Clinical, randomized, prospective study. Forty patients aged between 23 and 63 years with chronic peripheral vestibular disorders underwent 12 sessions of rehabilitation with visual stimuli using digital video disk (DVD) (experimental group) or Cawthorne-Cooksey exercises (control group). The Dizziness Handicap Inventory (DHI), dizziness analog scale, and the sensitized Romberg static balance and one-leg stance tests were applied before and after the intervention.

**Results:**

Before and after the intervention, there was no difference between the experimental and control groups (*p* > 0.005) regarding the findings of DHI, dizziness analog scale, and static balance tests. After the intervention, the experimental and control groups showed lower values (*p* < 0.05) in the DHI and the dizziness analog scale, and higher values (*p* < 0.05) in the static balance tests in some of the assessed conditions.

**Conclusion:**

The inclusion of visual stimuli by digital images on vestibular and body balance rehabilitation is effective in reducing dizziness and improving quality of life and postural control in individuals with peripheral vestibular disorders.

## Resumo

**Introdução:**

Estímulos visuais podem induzir a adaptação vestibular e recuperar o equilíbrio corporal.

**Objetivo:**

Verificar o efeito de estímulos visuais por imagens digitais na reabilitação do equilíbrio corporal de vestibulopatias periféricas.

**Método:**

Estudo clínico, randomizado, prospectivo. Quarenta pacientes com vestibulopatia periférica crônica e idade entre 23 e 63 anos foram submetidos à 12 sessões de reabilitação com estímulos visuais em DVD (grupo experimental) ou aos exercícios de Cawthorne-Cooksey (grupo controle). *Dizziness Handicap Inventory* (DHI), escala visual analógica de tontura e testes de equilíbrio estático de Romberg sensibilizado e de apoio unipodal foram aplicados antes e após a intervenção.

**Resultados:**

Antes e após a intervenção, não houve diferença entre os grupos experimental e controle (p > 0,005) no DHI, escala visual analógica e testes de equilíbrio estático. Após a intervenção, o grupo experimental e o controle apresentaram valores menores (p < 0,05) do DHI e da escala visual analógica de tontura e valores maiores (p < 0,05) nos testes de equilíbrio estático em algumas condições avaliadas.

**Conclusão:**

A inclusão de estímulos visuais por imagens digitais na reabilitação do equilíbrio corporal é eficaz na redução da tontura, na melhora da qualidade de vida e do controle postural de vestibulopatias periféricas.

## Introduction

Postural control is the ability to keep one's balance in relation to gravity, maintaining or adjusting the position of the body mass center on the support base.^1^ Body balance is a complex process that depends on the integrity of vision, the vestibular system, the somatosensory system, central coordination, and muscular adjustments.^2^ Failures in any of these systems can cause dizziness or vertigo.^3^

Dizziness is a feeling of body balance disorder; it can be defined as a movement delusion or hallucination, a sensation of spatial disorientation, either of rotational type (dizziness), or non-rotational, such as instability, imbalance, and visual distortion. It is a common symptom, present in more than 10% of the population; it can be triggered by primary or secondary vestibular system dysfunction^4^ and, in approximately 85% of cases, the dizziness originates from the vestibular system.^5^

Vestibular rehabilitation exercises have been proposed in order to reduce the dizziness symptoms and improve body balance of individuals with vestibular dysfunction. There is moderate to strong evidence that vestibular rehabilitation is a safe and effective procedure in unilateral peripheral vestibular dysfunction; however, there is insufficient evidence to differentiate the results of different protocols from each other.^6^

Vestibular rehabilitation is based on repetitive exercises, associated with a change in habits and clarification of the dizziness symptoms. It is a physiological therapy method, which aims to stimulate the vestibular system and maximize the neuroplasticity of the central nervous system, accelerating and stimulating the natural mechanisms of compensation, adjustment, replacement, and habituation by promoting the restoration of the body balance of patients with dizziness.^7^, ^8^

Vestibular rehabilitation aims to modify the postural control system through repeated exposure to conflicting stimuli under different conditions. The exercises are aimed at reducing dizziness and body instability, increasing stabilization of visual and postural control in order to improve competence and well-being in daily activities.^9^, ^10^, ^11^

The first successful vestibular rehabilitation exercises protocol described in the literature was developed to treat patients with dizziness and cerebral concussion or submitted to labyrinthectomy, in order to accelerate the recovery by moving the eyes, head, and body.^12^, ^13^, ^14^, ^15^ According to a systematic review, this is the most widely used protocol in vestibular rehabilitation clinical trials,^16^ but it lacks specific exercises for simultaneous stimulation of proprioceptive and visual sensory information, support base modification, and other motor components.^17^

The repetitive movement of images in the retina through the combination of visual fixation exercises and head movements, with targets moving in the opposite direction to the movements of the head, and optokinetic training with the patient in the standing position while exposed to different sensory conditions can induce adaptation of vestibular responses and assist in the recovery of postural control. In these situations, the brain decreases retinal image slip by increasing the gain of the vestibular-ocular reflex, establishing the symmetry of the optokinetic nystagmus and decreasing visual dependence.^17−19^

A repetitive optovestibular stimulation program, consisting of sessions in the clinic with positional exercises, ocular pursuit, optokinetic stimulation, and rotational and caloric tests, was developed in order to readjust the vestibular function, producing habituation, adaptation, and facilitating body balance recovery.^20^ In 250 patients with vertigo and other types of dizziness treated with this procedure, it was observed that 26% of the patients became asymptomatic, 51.2% improved, 20% remained unchanged, and 2.8% worsened.^21^

The literature describes favorable results in patients with vestibular disorders after being submitted to optokinetic stimulation using a rotating drum with alternating vertical white and black stripes,^22^ with a Barany drum,^23^ and through computer optokinetic stimulation accompanying texts on a screen.^24^

Using a device that projects optokinetic stimuli on the walls, ceiling, and floor in a completely dark room, patients with labyrinthine hypofunction showed – after the stimulation – increased frequency, regulation of the slow-phase velocity of optokinetic nystagmus, and improvement in posturography parameters^18^; 80% of 75 sailors resistant to the use of preventive medication for motion sickness had no episodes of vomiting when traveling by ship.^25^ Also, patients with instability due to unilateral chronic peripheral vestibular dysfunction exhibited an improvement in balance and decreased visual response to environmental movements and situations of vestibular–visual conflict.^26^

In patients with chronic vestibular disorders, a protocol that included customized exercise programs for functional disability, including exposure at the clinic to environmental rotational images along with daily optokinetic stimulation at home with a digital video disk (DVD), resulted in improved postural stability and reduced the dizziness related to visual stimulation.^27^ The result of the optokinetic stimulation with this high-technology equipment was similar to the DVD stimulation with or without supervision; however, it was observed that 55% of the unsupervised patients did not complete the training program, compared to 10% of those who were supervised.^28^

It was concluded that the cost of equipment and the number of sessions at the clinic hindered the introduction of this program in daily practice, whereas the DVD with visual stimuli is an inexpensive and easy-to-manage tool to include optokinetic stimulation in vestibular rehabilitation programs; furthermore, supervision of the rehabilitation exercises helps with adherence, promotes the acquisition of postural stability, and improves patients’ emotional status.^19^, ^29^

Considering the benefits of optovestibular stimulation in body balance rehabilitation sessions carried out at the clinic and the importance of the stimulus repetition for vestibular adaptation, substitution of sensorimotor strategies, and the necessity for habituation to occur, a controlled form of training was proposed to the neurotological patient, that included a DVD with ocular fixation, slow ocular pursuit, and saccadic and optokinetic stimuli to accelerate the vestibular compensation process, which easily could be practiced every day at home, interactively, and cost-effectively.^30^

The aim of this study was to evaluate the effect of including visual stimuli through digital images in body balance rehabilitation of patients with peripheral vestibular disorders.

## Methods

This clinical, randomized, prospective cohort study was conducted from 2010 to 2013, after approval by the Research Ethics Committee of the institution under protocol No. 1016/10. All patients received information about the study and its objectives through an explanatory letter and signed the informed consent before the start of the investigation.

The sample consisted of 40 patients, randomly assigned to the experimental and control groups, following a randomized table prepared by uniform distribution, performed using SPSS version 19.0.

As inclusion criteria, male and female patients were selected, aged 18–64 years, complaining of dizziness for at least three months, reporting at least one dizziness episode a month, with a medical diagnosis of chronic peripheral vestibular disease, and who could use the DVD equipment in their homes.

Patients with diagnoses of benign paroxysmal positional vertigo and Meniere's disease, medical history or signs of central nervous system disorders and/or psychiatric disorders, uncontrolled hypertension and diabetes, incapacity to understand and follow simple verbal commands, inability to independently remain in the standing position, severe visual impairment or visual impairment not compensated by corrective lenses, orthopedic disorders that resulted in movement limitation or use of lower-limb prostheses, use of anti-dizziness medication, report of previous body balance rehabilitation in the last six months, absence in three consecutive sessions, or failure to follow the guidelines proposed in this investigation were excluded.

Individuals who did not meet the inclusion criteria of this study were referred to undergo vestibular rehabilitation outside the research protocol. The same therapist provided the recommendations and carried out exercise monitoring in both groups, in addition to evaluating the patients before and after the intervention.

Before the intervention, in order to assess vestibular system function and its interrelation with the central nervous system, patients were submitted to otorhinolaryngological examination; clinical history; application of the Brazilian version^31^ of the Dizziness Handicap Inventory (DHI)^32^; visual analog scale of dizziness^33^; assessment of static balance using the sensitized Romberg static balance and one-leg stance tests^34^, ^35^; and vestibular system functional assessment.^11^, ^36^, ^37^

The DHI was used to assess the impact of the effects caused by dizziness on quality of life before and after the intervention. The DHI consists of 25 questions: seven relate to physical aspects, nine to the emotional domain, and nine to the functional effects. The patients answered “yes,” “sometimes,” or “no” to each question, corresponding to 4, 2, or 0 points, respectively. The total score ranged from 0 to 100 points; with a maximum score of 28 points for the physical aspects, 36 points for the emotional aspects, and 36 points for the functional aspects. The DHI analysis classified the impact as mild when the score was between 0 and 30; moderate when between 31 and 60, and severe when between 61 and 100 points.^38^ Significant improvement after the treatment was considered when the difference between the scores before and after the intervention was higher than 18 points.^32^

The visual analog scale of dizziness was used to measure the intensity of pain before and after the intervention, according to the weight and the patient's classification of severity of his/her sensation of dizziness, by scoring on a ruler ranging from 0 to 10, with 0 being the lowest level of dizziness and 10 the highest.^33^

Evaluation of static body balance using the sensitized Romberg static balance and one-leg stance tests^34^, ^35^ was used to assess the performance by measuring the time in seconds during which the patient was able remain in the stipulated position, on stable and unstable surfaces with open and closed eyes, before and after the intervention. In the sensitized Romberg test, the patient was instructed to put one foot in front of the other in order to establish a straight line, with the dominant limb forward and the non-dominant one backward, and then with the non-dominant limb forward and the dominant one backward; in the one-leg stance test, the dominant and non-dominant lower limbs were assessed by asking the patient to raise one leg, bending the knee. The time was counted with a stopwatch up to a maximum of thirty seconds or until the patient lost his/her balance, displaced the supporting foot, touched the other leg and put foot on the floor, or opened his/her eyes during the closed-eye condition three times in each of the tests; the best time of the three attempts was considered for the analysis.

Regardless of the protocol, vestibular rehabilitation was carried out at the clinic for six weeks in individual sessions, twice a week, each session lasting 40 min (total of 12 sessions). Patients were informed about all stages of treatment, the importance of performing daily exercises, and the possibility of increased dizziness, especially in the early stages of rehabilitation. They were also instructed to maintain their usual activities and to perform the exercises once a day at home, noting symptom evolution on a daily basis.

The control group used the Cawthorne-Cooksey protocol,^15^ consisting of eye, head, and trunk exercises. At the clinic, the exercises were introduced and modified according to the patient's capacity and evolution, absence of symptoms, safety, and ease to perform the movements. At home, the exercises were repeated every day under the same sensory conditions of the last session performed at the clinic.

The experimental group used a DVD with ocular fixation stimulus protocol, slow ocular pursuit, and saccadic and optokinetic movements,^30^ developed using Adobe Flash Professional CS5 software, Corel Draw X5, Adobe Premier Pro CS5, Adobe Photoshop CS5, and Nero Vision. The DVD's initial screen lists the 14 exercises and the following screens show the stimuli:

1. Ocular fixation stimulus: an image of a lamp changes color every two seconds in the center of a black screen, for three minutes.
2. Saccadic stimulus: an image of a white lamp randomly changes position every 1.5 s on a black screen, for 3 min.
3. Slow ocular pursuit stimulus: an image of a red insect in random movement on a green and blue screen for 3 min.
4. Optokinetic stimulus: an image of blue vertical bars moving from the right to the left on a white screen, with a velocity of 0.70 Hz, for 1 min.
5. Optokinetic stimulus: an image of blue vertical bars moving from the left to the right on a white screen, with a velocity of 0.70 Hz, for 1 min.
6. Optokinetic stimulus: an image of black horizontal bars moving from top to bottom on a white screen, with a velocity of 0.60 Hz, for 1 min.
7. Optokinetic stimulus: an image of black horizontal bars moving from bottom to top on a white screen, with a velocity of 0.60 Hz, for 1 min.
8. Optokinetic stimulus: an image of colored letters moving from left to right on a black screen, with a velocity of 0.80 Hz, for 1 min.
9. Optokinetic stimulus: an image of colored letters moving from right to left on a black screen, with a velocity of 0.80 Hz, for 1 min.
10. Optokinetic stimulus: an image of colored letters moving from top to bottom on a black screen, with a velocity of 0.80 Hz, for 1 min.
11. Optokinetic stimulus: an image of colored letters moving from bottom to top on a black screen, with a velocity of 0.80 Hz, for 1 min.
12. Optokinetic stimulus: an image of red bands in a convergent movement in relation to a central point on a white screen, for 1 min.
13. Optokinetic stimulus: an image of red bands in a concentric movement in relation to a central point on a white screen, for 1 min.
14. Optokinetic stimulus: simulation of a trajectory on a tree-lined road, for 1 min.

At the clinic, DVD stimuli were projected on a screen hung on the wall in a dark room and the patient in the experimental group was positioned 2 m away. At every session, the 14 exercises were performed by modifying the sensory conditions, according to the patient's skill and progress, considering the patient's ease to perform the exercises, as well as symptom absence.

1. Sitting, without moving the head;
2. Sitting, performing head-rotation movements to both sides and then flexion and extension movements;
3. Bouncing on a Swiss ball without moving the head;
4. Bouncing on a Swiss ball, performing head-rotation movements to both sides and then flexion and extension movements;
5. Standing on a stable surface, without moving the head;
6. Standing on a stable surface, making head-rotation movements to both sides and then flexion and extension movements;
7. Standing on density-33 foam, without moving the head;
8. Standing on the foam, performing head-rotation movements to both sides and then flexion and extension movements;
9. Marching in place on stable surface, without moving the head;
10. Marching in place on stable surface, performing head-rotation movements to both sides and then flexion and extension movements;
11. Marching in place on the foam, without moving the head;
12. Marching in place on the foam, performing head-rotation movements to both sides and then flexion and extension movements;
13. Walking three steps forward and then backwards, without moving the head;
14. Walking three steps forward and then backwards, performing head-rotation movements to both sides and then flexion and extension movements.

At home, the stimuli were reproduced by a DVD player and were viewed on a TV in a dark room with the patient safely positioned one meter away. The 14 exercises were repeated every day under the same sensory conditions of the last session performed at the clinic.

After the intervention, all patients in both groups were submitted again to DHI, visual analog scale of dizziness, and evaluation of static balance through the sensitized Romberg test and one-leg stance tests to measure the performance after rehabilitation, and were referred to the otorhinolaryngologist for directions on continuity of care.

The results were submitted to statistical analysis using SPSS, version 21.0. The significance level for the statistical tests was set at 5% (*a* = 0.050). To describe the sample, categorical variables were shown as absolute (*n*) and relative (%) frequencies; scalar data through mean, standard deviation, and minimum and maximum values. The comparison between the experimental and the control group was carried out by applying the likelihood ratio test for categorical variables; the Mann–Whitney test was used for scalar variables. The intra-group comparison before and after the intervention was carried out by Wilcoxon signed-rank test.

## Results

During the data collection period, a total of 164 individuals were referred to the vestibular rehabilitation waiting list, of whom 84 were excluded by telephone contact (they did not have dizziness, had a disabling medical condition, or did not want to undergo the treatment), 38 did not meet the selection criteria, and two were excluded during the intervention, one due to headache that made it impossible to perform the exercises and the other due to nonattendance. The final sample consisted of 40 patients randomized between the control group (*n* = 20) and the experimental group (*n* = 20).

The diagnosis established by the otorhinolaryngologist and the respective numbers of cases in the experimental group were as follows: metabolic labyrinthine disorders (seven), vestibular neuritis (four), labyrinthine trauma (four), cervical syndrome (two), ototoxicity (one), motion sickness (one), and post-stapedectomy labyrinthine disorder (one); in the control group: metabolic labyrinthine disorder (nine), vestibular neuritis (five), cervical syndrome (three), labyrinthine trauma (two), and motion sickness (one).

Table 1 shows the sample distribution before the intervention according to age, gender, findings at the functional assessment of the vestibular system, duration, frequency, and time of dizziness onset. There was no statistically significant difference between the groups regarding age, gender, findings at the functional assessment of the vestibular system, duration, frequency, and the time of dizziness symptom onset.

**Table 1:** Demographic and clinical characteristics of the patients in the experimental group and the control group before the intervention.

Characteristic	Experimental group (*n* = 20)	Control group (*n* = 20)	*p*
*Mean age (minimum–maximum)*	45.85 (23–63)	51.85 (32–63)	0.278^a^

*Gender, n (%)*			
Male	6 (30%)	3 (15%)	0.256^b^
Female	14 (70%)	17 (85%)	

*Vestibular assessment findings, n (%)*			
Normal vestibular function	7 (35%)	9 (45%)	
Directional preponderance	0 (0%)	1 (5%)	
Bilateral hyperreflexia	5 (25%)	4 (20%)	0.656^b^
Unilateral hypofunction	7 (35%)	6 (30%)	
Bilateral hypofunction	1 (5%)	0 (0%)	

*Dizziness duration, n (%)*			
Days	1 (5%)	1 (5%)	
Hours	4 (20%)	2 (10%)	0.532^b^
Minutes	4 (20%)	8 (40%)	
Seconds	11 (55%)	9 (45%)	

*Dizziness frequency, n (%)*			
Monthly	2 (10%)	2 (10%)	
Weekly	7 (35%)	12 (60%)	0.248^b^
Daily	11 (55%)	6 (30%)	

*Time of dizziness onset, n (%)*			
1 to 2 years	5 (25%)	5 (25%)	
2 to 4 years	6 (30%)	9 (45%)	0.549^b^
More than 5 years	9 (45%)	6 (30%)	

a Mann–Whitney test.

b likelihood ratio test.

Table 2 shows the intra-group and inter-group comparison of the values in the DHI, and the visual analog scale of dizziness in the experimental and the control groups before and after the intervention. When comparing the experimental group and the control group before the intervention, there was no significant difference in the values of the DHI and the visual analog scale of dizziness; after the intervention, the values of the DHI and the visual analog scale of dizziness were significantly lower in both groups, without significant differences between them. Difference greater than 18 points in the total DHI score was found in 75% of cases in the experimental group and in 50% of the control group cases.

**Table 2:** Intragroup and intergroup comparison of the values of the Dizziness Handicap Inventory and visual analog scale of dizziness in the experimental group and the control group, before and after the intervention.

Testes	Experimental group			Control group				
	Before	After	*p*^1^	Before	After	*p*^1^	*p*^2^	*p*^3^
	Mean/SD (min–max)			Mean/SD (min–max)				
DHI – total	51.30/26.54 (6–100)	24.60/23.13 (0–74)	<0.001^a^	47.90/18.78 (18–98)	24.20/14.70 (2–56)	0.000^a^	0.715	0.056
DHI – physical	16.80/7.06 (4–28)	6.80/6.97 (0–22)	<0.001^a^	15.50/5.19 (8–28)	8.30/5.92 (0–24)	0.000^a^	0.445	0.333
DHI – emotional	16.10/11.02 (0–36)	8.10/9.64 (0–30)	<0.001^a^	14.80/10.63 (0–36)	6.60/7.40 (0–24)	0.002^a^	0.776	0.681
DHI – functional	18.40/10.40 (0–36)	9.70/9.50 (0–28)	<0.001^a^	17.60/7.72 (4–34)	9.30/5.48 (0–20)	0.000^a^	0.714	0.479
Visual analog scale of dizziness	7.45/2.04 (3–10)	3.15/2.94 (0–8)	<0.001^a^	6.40/1.67 (4–10)	3.90/2.00 (0–7)	0.001^a^	0.071	0.293

SD, standard deviation; min, minimum value; max, maximum value; DHI, Dizziness Handicap Inventory; *p*^1^, intragroup comparison; *p*^2^, intergroup comparison before the intervention; *p*^3^, intergroup comparison after the intervention.

a Significant values. ^1^ Wilcoxon signed-rank test. ^2,3^ Mann–Whitney test.

Table 3 shows the intragroup and intergroup comparison of values in seconds while patients in the experimental group and the control group remained in the sensitized Romberg test position before and after the intervention. Comparing the experimental group and the control group before and after the intervention, there was no significant difference between the values of the sensitized Romberg test in all evaluated sensory conditions. After the intervention, in the experimental group, the values were significantly higher with eyes closed on stable and unstable surfaces, with the right foot and left foot forward; in the control group, the values were significantly higher with closed eyes on a stable surface with the left foot forward and on an unstable surface with the right foot and left foot forward.

**Table 3:** Intragroup and intergroup comparison of the values in seconds during which patients from the experimental and the control groups remained in the sensitized Romberg static balance test position before and after the intervention.

Testes	Experimental group			Control group				
	Before	After	*p*^1^	Before	After	*p*^1^	*p*^2^	*p*^3^
	Mean/SD (min–max)			Mean/SD (min–max)				
SS OE left	27.90/6.55 (3–30)	29.75/1.12 (25–30)	0.109	28.75/5.59 (5–30)	28.00/6.37 (5–30)	0.317	0.311	0.515
SS OE right	27.20/7.58 (2–30)	29.75/1.12 (25–30)	0.109	25.60/9.19 (5–30)	28.40/5.30 (8–30)	0.138	0.653	0.515
SS CE left	17.25/12.03 (0–30)	23.70/10.24 (2–30)	0.005^a^	16.65/11.78 (0–30)	20.60/11.50 (1–30)	0.020^a^	0.813	0.558
SS CE right	13.20/11.32 (0–30)	21.45/10.58 (2–30)	0.004^a^	17.15/12.44 (2–30)	20.45/10.99 (0–30)	0.244	0.260	0.619
US OE left	27.95/5.34 (10–30)	29.20/3.58 (14–30)	0.285	26.50/8.90 (0–30)	28.50/6.71 (0–30)	0.180	0.879	0.971
US OE right	27.80/6.20 (6–30)	29.60/1.79 (22–30)	0.109	27.35/7.33 (1–30)	28.55/6.03 (3–30)	0.197	0.948	0.554
US CE left	12.05/11.66 (0–30)	20.35/11.60 (1–30)	0.006^a^	8.70/8.63 (0–30)	15.80/12.78 (0–30)	0.023^a^	0.634	0.192
US CE right	11.60/11.09 (0–30)	17.45/11.53 (1–30)	0.001^a^	8.25/8.59 (0–30)	14.60/12.12 (0–30)	0.005^a^	0.423	0.381

SD, standard deviation; min, minimum value; max, maximum value; SS, stable surface; US, unstable surface; OE, open eyes; CE, closed eyes; *p*^1^, intra-group comparison, *p*^2^, inter-group comparison before the intervention; *p*^3^, inter-group comparison after the intervention.

a Significant values. ^1^ Wilcoxon signed rank test. ^2,3^ Mann–Whitney test.

Table 4 shows the intragroup and intergroup comparison of the values in seconds while patients in the experimental group and the control group remained in position during the one-leg stance test before and after the intervention. When comparing the experimental group and the control group before and after the intervention, there was no significant difference between the values in all assessed sensory situations. After the intervention, in the experimental group, the values were significantly higher with open eyes on a stable surface with right foot support, unstable surface with right and left foot support, eyes closed on a stable surface with right and left foot support, and on an unstable surface with right foot support. In the control group, the values were significantly higher with eyes closed on stable and unstable surfaces with right foot support.

**Table 4:** Intragroup and intergroup comparison of the values in seconds during which patients from the experimental and the control groups remained in the one-leg stance test position before and after the intervention.

Testes	Experimental group			Control group				
	Before	After	*p*^1^	Before	After	*p*^1^	*p*^2^	*p*^3^
	Mean/SD (min–max)			Mean/SD (min–max)				
SS OE left	24.20/10.49 (3–30)	25.895/8.27 (7–30)	0.068	26.00/9.00 (2–30)	26.55/7.75 (3–30)	0.799	0.929	0.711
SS OE right	21.55/11.34 (0–30)	24.25/9.25 (0–30)	0.017^a^	25.35/8.95(3–30)	27.25/7.12 (2–30)	0.611	0.276	0.242
SS CE left	6.75/7.50 (1–30)	9.90/7.75 (2–30)	0.021^a^	6.85/5.20 (0–20)	10.85/10.59 (0–30)	0.154	0.369	0.849
SS CE right	6.35/6.76 (0–25)	10.35/9.41 (0–30)	0.028^a^	5.00/3.29 (0–13)	8.20/7.14 (0–30)	0.046^a^	0.785	0.515
US OE left	22.05/11.93 (2–30)	25.10/9.04 (3–30)	0.012^a^	21.75/10.85 (1–30)	23.40/9.05 (1–30)	0.505	0.744	0.333
US OE right	20.65/2.25 (0–30)	24.65/9.35 (0–30)	0.012^a^	23.70/10.48 (1–30)	24.25/9.53 (2–30)	0.483	0.454	0.833
US CE left	6.35/6.69 (0–23)	4.95/3.89 (0–14)	0.459	4.45/3.40 (0–14)	6.55/5.73 (0–21)	0.146	0.828	0.446
US CE right	5.50/5.57 (0–20)	7.25/5.89 (0–19)	0.013^a^	3.25/2.12 (0–8)	4.55/2.16 (0–8)	0.027^a^	0.511	0.263

SD, standard deviation; min, minimum value; max, maximum value; SS, stable surface; US, unstable surface; OE, open eyes; CE, closed eyes; *p*^1^, intra-group comparison, *p*^2^, inter-group comparison before the intervention; *p*^3^, inter-group comparison after the intervention.

a Significant values. ^1^ Wilcoxon signed rank test. ^2,3^ Mann–Whitney test.

## Discussion

Patients from the experimental and the control groups before the intervention were similar regarding gender, age, DHI, the analog scale of dizziness, the findings of functional assessment of vestibular function, the sensitized Romberg and one-leg stance tests, as well as the duration, frequency, and time of dizziness onset, indicating sample homogeneity.

Most randomized clinical trials with patients with vestibular disorders have shown zero or low study withdrawal rates and have not reported adverse events.^6^ The rate of withdrawal (4.76%, two cases), both in the control group, may be considered as within the expected range.

In this study, DVD was the media type chosen to introduce fixation, slow ocular pursuit, and saccadic and optokinetic stimuli in body balance rehabilitation, as it is affordable, easy to use, and inexpensive, which favors its use in clinical practice, factors that were also highlighted in another study that supported the utility of incorporating optokinetic stimuli in vestibular rehabilitation programs.^19^, ^29^

In a systematic review of vestibular rehabilitation with patients with vestibular disorders, the primary endpoints considered were those found in subjective symptom assessments, such as the visual analog scale of dizziness, vertigo scale, and the measurement of vertigo frequency and intensity; and as secondary outcomes, those observed in the objective assessment in the Romberg test, posturography, dynamic gait index, and DHI, among others.^6^ The present study assessed the subjective and objective aspects of dizziness through the visual analog scale of dizziness, DHI, sensitized Romberg test, and one-leg stance test, which are practices recommended by the Barany Society *Ad Hoc* Committee on Vestibular Rehabilitation Therapy, among others.^39^

The comparison between the experimental and control groups before and after the intervention showed similar values at the visual analog scale of dizziness, but both groups showed lower values after treatment, indicating a reduction in dizziness intensity. We did not find reports in the literature on the use of the visual analog scale of dizziness to measure the effect of rehabilitation of body balance with the inclusion of visual stimuli through digital images; however, improvement of symptoms by means of optokinetic stimuli has been reported in several publications.^18^, ^21^, ^22^, ^23^

Total DHI before treatment showed a moderate effect of symptoms on quality of life^38^ in the experimental group (mean of 51.30 points) and in the control group (mean of 47.90 points); these results are similar to those of other studies that found means of 54.40 points^40^ and 52.27 points.^41^

When comparing the experimental and the control groups before and after the intervention, the DHI values were similar. After the intervention, both groups had lower total DHI values and DHI values related to the physical, functional, and emotional aspects; 75% of cases in the experimental group and 50% in the control group showed a difference greater than 18 points in total DHI, which was considered indicative of improved quality of life.^32^ A study that also employed optokinetic stimuli considered it unexpected not to have found significant differences in DHI scores after rehabilitation, despite having observed body balance improvement at the computer dynamic posturography.^26^

When comparing the experimental and the control groups before and after the intervention, the values of the sensitized Romberg and one-leg stance tests under the assessed sensory conditions were comparable, similar to those previously reported in patients with vestibular dysfunction who underwent training at home, with or without additional physical rehabilitation.^42^ After the intervention, those with higher values in both groups showed better postural control. The present authors found no studies that had measured the effect of including visual stimuli through digital images in body balance rehabilitation of patients with vestibular disorders using these tests. There have been reports of body balance improvement with different protocols of optokinetic rehabilitation at posturography,^18^, ^24^, ^26^, ^29^ in which the repetitive optokinetic exposure had an effect on the adaptive mechanisms, resolving the optokinetic and vestibulo-ocular reflex asymmetries, improving postural control in situations where vision is inaccurate and conflicts with vestibular and somatosensory information.

At the sensitized Romberg test with open eyes on stable and unstable surfaces with the right foot and left foot forward, the intra-group comparison in the experimental group and in the control group showed no improvement in body balance, possibly because most patients in both groups showed maximum (30 s) or near-maximum values before and after the intervention, which also was observed previously.^42^

In the experimental group, the better performance in ten of the 16 sensory conditions assessed by the sensitized Romberg and one-leg stance tests and, in the control group, in only five, might suggest that the experimental group was able to improve the vestibular–visual–proprioceptive interaction more than the control group. The hypothesis that repeated exposure to visual stimuli stimulates neuronal plasticity by inducing adaptive mechanisms that reduce the magnitude of visual dependence on postural responses^19^ might explain the difference in performance between the groups.

This research showed that regardless of the rehabilitation protocol used, the results were favorable regarding body balance, decrease in symptoms, and improved patient quality of life. A systematic review emphasized that vestibular rehabilitation is effective, but there is no evidence that one protocol is superior to another.^6^ Similar outcomes with the two interventions corroborate the assertion that the practice of physical exercises for eyes, head, and body help with vestibular compensation and body balance recovery.^43^, ^44^

The homogeneity of the randomly selected sample groups, supervised sessions at the clinic for the two groups, explanations and sequential training of the exercises, customization according to symptom evolution, and adherence to treatment in the present study are noteworthy. The study was not double-blinded, but the one-leg stance test, in addition to the two other outcome measures used in this study, the DHI and the sensitized Romberg test, are considered objective measures.^6^ Although the exercises were not chosen according to the different symptoms and functional disabilities of each patient, the therapeutic result can be considered favorable in both groups.

The results of the experimental group, similar to those of the Cawthorne-Cooksey protocol, were employed in the control group, whose efficiency is described in the literature,^12^, ^13^, ^14^, ^15^ indicating that the inclusion of visual stimuli by digital images in body balance rehabilitation in patients with peripheral vestibular disorders was also effective, thus providing a new option for rehabilitation professionals. Further studies, including other evaluation procedures, are recommended to confirm these findings utilizing visual stimuli by digital images in body balance rehabilitation of patients with peripheral vestibular disorders.

## Conclusion

The inclusion of visual stimuli by digital images in body balance rehabilitation is effective in reducing dizziness, improving quality of life and postural control in patients with peripheral vestibular disorders.

## Conflicts of interest

The authors declare no conflicts of interest.
